# Children’s Internalizing Symptoms and Well-Being: The Role of Parental Anxiety and Health-Related Quality of Life

**DOI:** 10.3390/pediatric18040089

**Published:** 2026-07-06

**Authors:** Vasiliki Georgousopoulou, Georgios Manomenidis, Aspasia Serdari

**Affiliations:** 1 Department of Nursing, Democritus University of Thrace, 681 00 Alexandroupolis, Greece; gmanomen@nurs.duth.gr; 2 Department of Child and Adolescent Psychiatry, Democritus University of Thrace, 681 00 Alexandroupolis, Greece; aserntar@med.duth.gr

**Keywords:** children, quality of life, PedsQL, parental anxiety, internalizing symptoms, family factors, parent-proxy report

## Abstract

Background. Children’s health-related quality of life (HRQoL) has been associated with both individual and family-related factors, including internalizing symptoms and parental psychological well-being. Although previous research has highlighted the role of parental mental health, evidence from non-clinical community samples remains limited, particularly when parent-proxy reports are used. Methods. A cross-sectional study was conducted among 242 parents of children aged 8–12 years in Northern Greece. Parents completed proxy measures of children’s HRQoL and internalizing symptoms, as well as self-reported measures of their own HRQoL and anxiety. Nonparametric tests were used for bivariate analyses, and multiple linear regression was applied to identify independent predictors of children’s HRQoL. Results. Higher parental mental HRQoL was positively associated with children’s HRQoL (ρ = 0.213, *p* = 0.031), while parental anxiety (trait anxiety: ρ = −0.204, *p* = 0.004; state anxiety: ρ = −0.314, *p* < 0.001) and parent-reported child internalizing symptoms (depression: ρ = −0.369, *p* < 0.001; anxiety: ρ = −0.322, *p* < 0.001) were negatively associated with HRQoL; however, in the multivariable model, only parental mental HRQoL (B = 0.344, *p* = 0.020) and parental education (B = −2.944, *p* = 0.044) remained significantly associated with parent-proxy child HRQoL, explaining 29.2% of the variance in children’s HRQoL (R^2^ = 0.292). Conclusions. The findings suggest that parent-proxy child HRQoL is associated with parental psychosocial functioning in this community-based sample. Parental mental HRQoL was the strongest independent correlate of parent-proxy child HRQoL. However, given the exclusive use of parent-proxy reports and the convenience-based sample, these findings should be interpreted cautiously, as shared method variance, rater-related effects, and limited generalizability may have contributed to the observed associations. Further multi-informant and longitudinal studies conducted in more diverse populations are warranted.

## 1. Introduction

Children’s quality of life (QoL) and parental proxy reporting are important for understanding children’s well-being and family functioning [1]. The World Health Organization defines QoL as “individuals’ perception of their position in life within the context of the culture and value systems in which they live, and in relation to their goals, expectations, standards, and concerns” [2]. As a multidimensional construct, QoL encompasses physical, emotional, social, and behavioral well-being [3]. Parental proxy reporting reflects the understanding that children’s QoL includes both subjective and observable dimensions. In cases where children are too young to report reliably on their own well-being, parents can provide valuable proxy information, which is essential for accurate assessment in clinical practice, research, and health policy [4]. Children’s QoL is associated with the interaction of internal and external factors. Internal protective factors, such as psychological resilience, emotional maturity, and self-esteem, contribute to their ability to respond to developmental and environmental challenges while maintaining satisfactory levels of well-being [5]. External factors, particularly the family environment and parenting responses to child anxiety may either strengthen or undermine children’s adaptive capacity, indirectly shaping anxiety and children’s appraisals of QoL [6]. In childhood, anxiety is frequently presented with prominent somatic complaints (e.g., abdominal pain, headache, nausea) without identifiable organic etiology and is also associated with avoidance (including school refusal), emotional distress, and impaired school functioning or academic performance [7]. Beyond categorical diagnoses, anxiety symptoms frequently co-occur with depressive symptoms and are increasingly conceptualized as part of a broader internalizing spectrum [8]. Internalizing symptoms (anxiety and depressive manifestations) are consistently associated with poorer self-reported QoL and greater functional impairment in both children and adolescents, as supported by recent reviews and longitudinal evidence [1]. Moreover, longitudinal research indicates robust links between internalizing symptoms and lower self-esteem during adolescence, with evidence of bidirectional associations [9,10]. The relationship between internalizing symptoms and QoL is embedded within the family context. Family-systems and ecological perspectives conceptualize the family as an interdependent system in which parents’ well-being and emotion-regulation capacities shape children’s emotional adjustment through everyday parenting processes and proximal interactions [11,12]. Evidence from meta-analytic and systematic review work further supports links between parental stress/well-being and child adjustment, underscoring the relevance of whole-family approaches when parental mental health difficulties are present [13,14]. Parenting processes that shape children’s emotion regulation, such as parental emotion socialization show small-to-moderate associations with child internalizing symptoms [15], while broader parenting patterns such as overparenting have been meta-analytically linked to offspring depression, anxiety, and internalizing symptoms [16]. Additionally, relational features within the family (e.g., differential treatment between siblings) have been associated with children’s internalizing and externalizing behavior in meta-analytic findings [17]. Together, this body of evidence supports a dyadic approach that considers parental anxiety and health-related quality of life (HRQoL) alongside children’s QoL and internalizing symptoms. Higher levels of parental stress, anxiety, and depression have been associated with poorer child and adolescent QoL, as well as with greater mental health difficulties, indicating that parental psychological distress is closely linked to children’s well-being outcomes [18]. In addition, parental mental health may influence, or be associated with, proxy ratings of children’s HRQoL, because parents experiencing distress may perceive their child’s health and functioning more negatively than the child does, especially in less observable domains, such as emotional, social, and school functioning, whereas agreement tends to be stronger for more visible domains such as physical functioning [19,20]. This is particularly important in chronic pediatric conditions, where parent–child discrepancies are common and parents often report lower HRQoL than children’s self-reports suggest, reflecting the complexity of proxy assessment rather than merely the child’s objective condition [4,20]. Moreover, cultural and contextual differences may further shape parental assessments, since sociocultural expectations, child-rearing norms, family roles, and beliefs about illness may influence how parents interpret and evaluate their child’s QoL. For example, evidence from the Hong Kong Chinese context suggests that culturally shaped expectations regarding academic performance, conformity, and parenting style may contribute to parent–child differences in QoL ratings [20]. Therefore, when interpreting parent-proxy HRQoL reports, it is important to consider not only the child’s clinical status, but also the parents’ own psychological burden, especially symptoms of anxiety and depression, as well as disease-specific and cultural particularities that may influence reporting patterns [4,18,19]. In the present study, children’s QoL was assessed using the Pediatric Quality of Life Inventory (PedsQL) 4.0 Generic Core Scales [21], a widely used and psychometrically validated instrument for measuring HRQoL in pediatric populations. The instrument was administered exclusively to parents (parent-proxy report) in order to ensure reliable data collection in younger age groups or in cases where children were unable to provide accurate responses. The use of parent-proxy reports is considered essential in such cases, as parents can provide valuable information regarding the child’s daily functioning and well-being, particularly in less observable domains such as emotional and psychosocial functioning [19]. Despite growing evidence, research gaps remain regarding non-clinical community samples, where subclinical anxiety and internalizing symptoms may still meaningfully relate to children’s QoL. Existing Greek studies have reported associations between parental internalizing symptoms and adolescents’ mental health and QoL [18], as well as elevated educational stress and anxiety-related symptoms among adolescents [22], further supporting the need for additional research in community samples. The aim of the study was to investigate the relationships between children’s QoL and anxiety levels in both children and their parents, based on parent-proxy reports, considering factors influencing daily functioning and emotional well-being. Accordingly, we hypothesized that (H1) higher parental anxiety would be associated with lower parent-proxy child HRQoL, (H2) poorer parental mental HRQoL would be associated with lower parent-proxy child HRQoL, and (H3) higher parent-reported child internalizing symptoms would be associated with lower parent-proxy child HRQoL.

## 2. Materials and Methods

### 2.1. Study Design and Participants

This study employed a descriptive cross-sectional design to investigate the relationships between children’s HRQoL and internalizing symptoms (anxiety and depression), as reported by parents, and parents’ own HRQoL and anxiety levels. Data collection was carried out between September and December 2025 in the region of Thrace, Northern Greece. A convenience sampling method was used. The study population consisted of 242 parents or primary caregivers of children aged 8–12 years residing in Thrace. Inclusion criteria required participants to (a) be the parent or primary caregiver of at least one child aged 8–12 years, (b) live in the same household as the child, and (c) be able to read and understand Greek. According to parental reports, families were excluded if the target child had a diagnosed intellectual disability, severe neurological disorder, psychotic disorder, or severe chronic medical condition that could substantially affect daily functioning and quality of life. A brief invitation describing the aims and procedures of the study was distributed via parents’ associations, inviting interested parents to participate. The questionnaire was administered in two equivalent formats: (a) a printed, self-administered paper version delivered hand-to-hand to parents and returned in sealed envelopes, and (b) an electronic version created in Google Forms and accessed via a secure link shared through the parents’ associations and social media. In both formats, parents completed proxy-report measures regarding their child (child HRQoL and internalizing symptoms), as well as self-report measures concerning their own HRQoL and anxiety. Participation was anonymous and voluntary. All returned questionnaires that met the inclusion criteria were considered valid and were included in the final analysis.

### 2.2. Measures

A battery of questionnaires was used to assess (a) demographic and family characteristics; (b) the Pediatric Quality of Life Inventory (PedsQL 4.0); (c) the Revised Child Anxiety and Depression Scale—25-item, Caregiver Version (RCADS-25-CG); (d) the Short Form Health Survey (SF-12); and (e) the State–Trait Anxiety Inventory (STAI).

### 2.3. Demographic Characteristics

The demographic characteristics questionnaire, developed for the study, included questions about gender, parental education, age, marital status and the presence of children with chronic illness.

### 2.4. The Pediatric Quality of Life Inventory (PedsQL)

Children’s HRQoL was assessed using the Pediatric Quality of Life Inventory (PedsQL 4.0 Generic Core Scales) [21]. PedsQL consists of 23 items and evaluates children’s QoL across 4 dimensions: (1) Physical Functioning (8 items): assesses the child’s ability to perform everyday physical activities; (2) Emotional Functioning (5 items): assesses the child’s emotional state and psychological well-being; (3) Social Functioning (5 items): assesses the child’s ability to interact with peers and participate in social activities; (4) School Functioning (5 items): assesses academic functioning and the impact of health on school performance. The instrument is designed for children and adolescents aged 2–18 years. Respondents rate each item on a 5-point Likert scale, from 0 (never) to 4 (almost always). For scoring, items are reverse-scored and linearly transformed to a 0–100 scale. Subscale scores are calculated by averaging the item scores within each dimension, with higher scores indicating better quality of life.

Item responses were reverse-scored and linearly transformed to a 0–100 scale according to the official PedsQL scoring algorithm (0 = 100, 1 = 75, 2 = 50, 3 = 25, 4 = 0). This transformation is recommended by instrument developers to facilitate interpretation and comparability across studies and does not alter the statistical relationships among variables. The PedsQL has been translated, culturally adapted, and validated in Greek pediatric populations, and is appropriate for use in Greek-speaking children and adolescents. Permission for use was obtained from the original developers via email [23].

### 2.5. The Short Form Health Survey (SF-12)

The Short Form Health Survey (SF-12), developed by Ware et al. [24] was used to assess HRQoL in parents. The SF-12 consists of 12 items covering eight health domains: (1) Physical Functioning (PF): limitations in performing physical activities; (2) Role Physical (RP): role limitations due to physical health problems; (3) Bodily Pain (BP): intensity of pain and its impact on daily activities; (4) General Health (GH): overall self-perception of health; (5) Vitality (VT): energy and fatigue; (6) Social Functioning (SF): impact of physical and emotional health on social activities; (7) Role Emotional (RE): role limitations due to emotional problems; and (8) Mental Health (MH): symptoms of anxiety, depression, and overall psychological well-being. The SF-12 has been translated into Greek and standardized in the general population [25]. Responses are scored to produce two summary indices: the Physical Component Summary (PCS) and the Mental Component Summary (MCS). Scores range from 0 to 100, with higher scores indicating better health status.

### 2.6. The Revised Child Anxiety and Depression Scale—25-Item, Caregiver Version (RCADS-25-CG)

Symptoms of anxiety and depression in children, as reported by parents, were assessed using the Revised Child Anxiety and Depression Scale—25-item Caregiver version (RCADS-25-CG) [26]. The instrument consists of 25 items describing common anxiety- and depression-related behaviors and emotions. Caregivers are asked to indicate how often each statement has been true for their child using a 4-point Likert scale from 0 (Never) to 3 (Always). Item scores are summed to yield an overall internalizing symptom score, with higher scores indicating higher levels of anxiety and depressive symptomatology. The caregiver version used in the present study is the authorized Greek translation, adapted for Greek-speaking parents/caregivers and suitable for use in school-age children and adolescents [27].

### 2.7. The State–Trait Anxiety Inventory (STAI)

The State–Trait Anxiety Inventory (STAI) [28] was used to assess both situational and dispositional anxiety. It consists of 40 items divided into two 20-item subscales: the State Anxiety scale (SAI) and the Trait Anxiety scale (TAI). The State Anxiety scale evaluates how the respondent feels “right now, at this moment,” capturing transient, situational anxiety responses (e.g., tension, apprehension, nervousness). Responses are given on a 4-point Likert scale ranging from 1 (not at all) to 4 (very much so), with higher scores indicating higher state anxiety; total scores range from 20 to 80. The TAI assesses how the respondent feels in general, usually reflecting relatively stable individual differences in the tendency to experience anxiety across daily life situations. Items are rated on a 4-point Likert scale according to the frequency of the described feelings or behaviors from 1 (almost never) to 4 (almost always), with higher scores denoting greater trait anxiety; total scores range from 20 to 80.

### 2.8. Ethics Considerations

The study received ethical approval from the Scientific Council-Ethics and Deontology Committee (Approval No. DUTH/ΕHDΕ/50274/444/21-03-2025), and all participants were informed about the study’s objectives, as well as their right to withdraw at any time.

### 2.9. Statistical Analysis

Data were imported into IBM SPSS Statistics (version 24.0; IBM Corp., Armonk, NY, USA). Prior to analysis, the normality of continuous variables was assessed using the Kolmogorov–Smirnov test. Normality assumptions were not met. Because multiple linear regression assumes normality of residuals rather than normality of the observed variables, regression assumptions were evaluated using residual diagnostics, homoscedasticity, and multicollinearity indices; therefore, non-parametric procedures were applied for bivariate analyses. Continuous variables are presented as means (SD) to preserve interpretability on the original scale units and for comparability with the literature; however, because normality assumptions were not satisfied, between-group comparisons were performed using non-parametric (rank-based) tests, which do not require normally distributed data. Bivariate associations between the independent variables and PedsQL were examined using the Mann–Whitney U test (reported as Z where applicable), Spearman’s rank correlation coefficient (ρ), and the Kruskal–Wallis H test. Variables showing a significant association with PedsQL in the bivariate analysis were entered in a multiple regression model as predictors. Statistical significance was set at *p* < 0.05.

## 3. Results

Respondents included 242 parents, the majority of whom were female (88.8%), had tertiary education (41.7%) and were married (91.2%). Most participants were aged ≥41 years (63.2%), while 36.8% were aged ≤40 years. Participants aged exactly 40 years were included in the ≤40 years group. Regarding their children’s health status, the vast majority did not report any chronic illness (90.5%) Table 1.

Descriptive statistics for the scales and subscales (PCS, MCS, PedsQL, TAI, STAI, and RCADS) are presented in Table 2. In the present sample, both mean PCS (42.85 ± 5.59) and MCS (36.92 ± 6.98) were below the norm-based reference value of score 50 (Ware et al., [24], indicating low-to-moderate QoL. For PedsQL, total scores and subscale scores were above the reference score of norm-based 50, indicating better HRQoL [21]. The anxiety and depression subscales of the RCADS mean scores indicated moderate anxiety and depression (between cut-off scores of 38–44) [29].

Subsequently, bivariate analyses were conducted to examine associations between PedsQL and key variables (Table 3). Higher PedsQL scores were observed among parents with secondary education (mean rank = 143.20) compared with tertiary education (mean rank = 105.70), with the MSc/PhD group showing intermediate values (mean rank = 126.56). PedsQL also differed across age groups (<40 mean rank =132.89; >41: mean rank =114.87) and was higher among participants living with a partner/spouse (mean rank = 122.66) than among those without a partner/spouse (mean rank = 86.83). In addition, PedsQL was positively associated with MCS and negatively associated with TAI, SAI, RCADS Depression, and RCADS Anxiety.

Table 3 summarizes the bivariate analyses between children’s HRQoL and the study variables. Group differences according to categorical demographic variables were examined using the Mann–Whitney U or Kruskal–Wallis tests, whereas associations with continuous variables were assessed using Spearman’s rank correlation coefficient (ρ).

In the multiple linear regression predicting PedsQL total score, the overall model was significant, F(8, 233) = 12.01, *p* = 0.003, explaining 29.2% of the variance (R^2^ = 0.292; adjusted R^2^ = 0.268; SEE = 7.59; N = 242). Two predictors were statistically significant: parental education (B = −2.94, SE = 1.43, β = −0.254, *p* = 0.044) and MCS-12 (B = 0.34, SE = 0.14, β = 0.315, *p* = 0.020). Parent age, marital status, TAI mean, STAI mean, RCADS depression mean, and RCADS anxiety mean were not significant predictors (all *p* > 0.05). These findings are presented in Table 4. Multicollinearity was evaluated using variance inflation factors (VIFs) and tolerance. VIFs ranged from 1.38 to 3.17 (tolerance = 0.316–0.726), suggesting that multicollinearity was not problematic for the model. Residual diagnostics were examined through visual inspection of the normal Q–Q (or P–P) plot and the standardized residuals vs. fitted values plot; these checks did not indicate marked departures from approximate normality or homoscedasticity, supporting the adequacy of the linear regression assumptions.

## 4. Discussion

The study examined the associations between parental anxiety, children’s HRQoL, and internalizing symptoms in a non-clinical Greek sample of parents of children aged 8–12 years. In the multivariable regression model, parental education and parental mental HRQoL (SF-12 MCS) remained the only significant independent predictors of children’s HRQoL, underscoring the importance of considering parental psychosocial functioning when interpreting proxy reports of child HRQoL and when conceptualizing child well-being within a family systems framework.

On top of that, parental anxiety was inversely associated with children’s HRQoL. Thus, H1 received partial support: higher parental anxiety was associated with lower parent-proxy child HRQoL in the bivariate analyses, but this association did not remain significant after adjustment for parental mental HRQoL and other covariates. This pattern aligns with recent evidence that parental anxiety is linked to adverse emotional and behavioral outcomes in offspring, including internalizing problems, across heterogeneous study designs and populations [30]. However, parental anxiety did not retain significance in the multivariable model. One plausible explanation is construct overlap: broader parental mental HRQoL may capture the functional impact of psychological distress more comprehensively, thereby absorbing variance shared with symptom-specific indices such as anxiety. In addition, parental anxiety may exert indirect effects via parenting processes (e.g., accommodation, control, emotion socialization) and child emotion regulation, which were not directly measured here [15].

Parental mental HRQoL emerged as the strongest independent correlate of parent-proxy child HRQoL in the multivariable model. This finding supports H2, as parental mental HRQoL was positively associated with parent-proxy child HRQoL in the bivariate analysis and remained a significant independent predictor in the multivariable model, indicating that poorer parental mental HRQoL was associated with lower parent-proxy child HRQoL. However, this association should be interpreted cautiously given the exclusive reliance on parent-reported measures, which may have introduced common rater variance. This finding is consistent with the broader literature showing that parent-proxy HRQoL ratings are not interchangeable with child self-reports and that discrepancies between self- and proxy-reported pediatric HRQoL are common [19]. Importantly, parental psychological state can shape proxy ratings through at least two, non-mutually exclusive pathways: (a) a contextual family pathway, whereby parental mental well-being affects daily routines, emotional climate, and co-regulation opportunities that support children’s functioning; (b) a rater-related pathway, whereby parental distress influences the appraisal and reporting of child difficulties [19]. Because child HRQoL was assessed exclusively through parent-proxy reports, the present study cannot determine whether these associations primarily reflect children’s actual well-being, parents’ perceptions of their children’s well-being, or a combination of both. Therefore, the observed associations should be interpreted as relating to parent-perceived child HRQoL rather than objective child HRQoL.

Parent-reported child internalizing symptoms (RCADS anxiety and depression) showed strong bivariate associations with lower child HRQoL, consistent with contemporary evidence that internalizing problems are associated with poorer functioning and reduced quality of life in youth [1]. Thus, H3 received partial support, as both parent-reported child anxiety and depressive symptoms were associated with lower parent-proxy child HRQoL in the bivariate analyses, but neither remained an independent predictor in the adjusted model. The attenuation of these associations after adjustment for parental mental HRQoL should be interpreted cautiously. Although multicollinearity diagnostics did not indicate problematic levels (VIFs < 5), parental mental HRQoL, parental anxiety, and parent-reported child internalizing symptoms are conceptually related constructs that share common variance. Moreover, because both parental psychological characteristics and child outcomes were reported by the same informant, the observed pattern may partly reflect shared method variance and rater-related effects rather than indicating that child internalizing symptoms are less relevant to children’s HRQoL. Consequently, parental mental HRQoL should be interpreted as the strongest independent correlate of parent-proxy child HRQoL in the present sample, rather than evidence that it supersedes the contribution of child internalizing symptoms.

Beyond parental symptoms, multiple recent meta-analytic findings highlight the relevance of parenting processes for child internalizing outcomes. For example, parental depression has been meta-analytically associated with children’s internalizing and externalizing problems across cross-sectional and longitudinal designs [31], and clinically diagnosed parental depressive disorders are linked to elevated child internalizing/externalizing symptoms in recent meta-analytic work [32]. Parenting behaviors that shape children’s emotion regulation also show consistent associations with internalizing problems, including small-to-moderate links between parental emotion socialization practices and child internalizing symptoms [15]. Moreover, broader parenting patterns such as overparenting have been meta-analytically linked to offspring depression, anxiety, and internalizing symptoms [16]. These findings support interpreting parental mental HRQoL not only as a rater-related factor but also as an upstream family-level correlation that may be expressed through everyday parenting processes and children’s emotion regulation [33].

Sociodemographic factors also contributed to children’s parent proxy-HRQoL. Parental education predicted children’s parent-proxy HRQoL in both bivariate and adjusted analyses, with higher education associated with lower PedsQL scores. While this diverges from many socioeconomic-gradient findings, it may reflect differential expectations, greater sensitivity to psychosocial and school-related difficulties, or different thresholds for reporting impairment among more educated parents, particularly in parent-proxy designs [34,35]. It may also indicate residual confounding by unmeasured socioeconomic or workload-related factors. Marital status was associated with PedsQL in bivariate analyses but not in the adjusted model, suggesting that partnership status may operate through correlated psychosocial variables rather than independently predicting child HRQoL, consistent with recent evidence highlighting the role of family structure and parental wellbeing in shaping children’s HRQoL [36].

### 4.1. Implications for Family-Centered Assessment and Intervention

Within the context of this community-based sample, the results suggest a family-centered approach in pediatric and community settings, where parental mental well-being can be considered both a contextual correlation of child functioning and a factor influencing proxy appraisals of child HRQoL. These findings have important practical implications for pediatric and community health services. Routine screening of parental mental health, particularly aspects of mental HRQoL, could be incorporated into pediatric assessments to support a more comprehensive evaluation of child well-being. In addition, family-centered care models should be strengthened by systematically including parents in assessment and intervention processes, rather than focusing exclusively on the child. From an intervention perspective, brief, low-intensity, parent-focused programs (e.g., psychoeducation, stress management, and emotion regulation strategies) could be implemented in primary care, school, or community settings to support parental well-being and, indirectly, children’s quality of life. Furthermore, training healthcare professionals to recognize the potential influence of parental psychological status on parent-proxy reports may improve the interpretation of HRQoL assessments and clinical decision-making.

Finally, integrating parent components into preventive and early intervention strategies may be particularly beneficial in community populations, where subclinical symptoms may not meet diagnostic thresholds but still affect children’s everyday functioning and perceived quality of life.

These findings also highlight several priorities for future research, including the incorporation of child self-reports, the examination of parent–child agreement within the same sample, the investigation of developmental differences across childhood, and the use of longitudinal, multi-informant designs to better understand the mechanisms linking parental psychological functioning and children’s HRQoL.

### 4.2. Limitations and Strengths

Several limitations should be acknowledged. The cross-sectional design precludes causal inference and does not clarify directionality among parental mental HRQoL, parental anxiety, child internalizing symptoms, and child HRQoL. In addition, reliance on parent-proxy reporting for child outcomes introduces shared rater effects and potential proxy–self-report discrepancies, which are well documented in pediatric HRQoL research.

Parents’ own emotional status may have influenced their perceptions and ratings of their children’s health-related quality of life and internalizing symptoms, potentially introducing rater bias and affecting the reliability of proxy-reported outcomes. Furthermore, although children aged 8–12 years are generally able to provide reliable self-reports, child self-report measures were not included in the present study. Consequently, it was not possible to examine the level of agreement between children’s own perceptions and their parents’ proxy reports. Future studies should assess both parents and children within the same sample to evaluate parent–child agreement and to better distinguish between children’s actual well-being and potential influences of parental psychological characteristics on proxy ratings.

Furthermore, the use of convenience sampling limits the generalizability of the findings, as the sample may not be representative of the broader population. The voluntary nature of recruitment may have also introduced self-selection bias, potentially leading to the overrepresentation of parents who are more engaged, health-aware, or psychologically sensitized to their children’s wellbeing. As a result, the findings may reflect heightened reporting of difficulties or, conversely, increased awareness and responsiveness rather than objective differences in child HRQoL.

In addition, the predominance of mothers in the study sample limited our ability to examine potential differences between maternal and paternal proxy reports. Therefore, the findings may not be fully generalizable to fathers or to more gender-balanced caregiver populations.

Additionally, although key variables were included in the analysis, the possibility of residual confounding cannot be excluded, as other relevant factors such as parental workload, family functioning, or broader socioeconomic conditions were not directly measured. All measures were based on self-report, which may be subject to reporting bias, including social desirability and recall bias.

Moreover, the multivariable associations observed in the present study cannot distinguish between true family-level relationships and associations attributable to common rater variance. Future studies incorporating child self-reports and multiple informants are needed to disentangle these effects.

Finally, although all children were within the target age range of 8–12 years, exact child age was not available as an analyzable variable. Therefore, we could not examine whether parent-proxy child HRQoL or parent-reported internalizing symptoms differed across child age within this developmental period. Future studies should collect exact child-age data and investigate possible developmental differences in HRQoL and internalizing symptoms.

Despite these limitations, the study offers notable strengths. It addresses an important and underexplored area by examining the interplay between parental psychological factors and child HRQoL using validated instruments. The inclusion of both parental mental HRQoL and anxiety provides a more comprehensive understanding of parental influences, while the analytical approach allows for the examination of these relationships beyond simple bivariate associations. The findings contribute to the growing body of literature emphasizing the importance of family context in pediatric HRQoL and have potential implications for targeted interventions and family-centered care approaches. The RCADS-25 is a widely used and psychometrically sound brief screening instrument, with recent studies further supporting its suitability for community screening and normative benchmarking [37], thereby strengthening confidence in the measurement approach of the present study. At the same time, the use of a single informant, namely parents, reporting on both their own psychological characteristics and their child’s outcomes may introduce shared method variance, potentially inflating the observed associations across parent-reported measures.

Future studies should incorporate child self-report HRQoL, multi-informant assessments of internalizing symptoms, and direct measurement of family processes (e.g., parenting stress, emotion socialization, accommodation) to test plausible mechanisms supported by recent meta-analytic evidence [15]. Longitudinal designs would be especially valuable for clarifying temporal relations and informing targeted family-based interventions.

## 5. Conclusions

Overall, the findings highlight that parent-proxy child HRQoL is closely associated with the broader psychological and family context. These findings highlight the importance of considering parental psychosocial functioning when interpreting parent-proxy assessments of children’s HRQoL, recognizing that parents may act not only as informants but also as contributors to how their children’s well-being is perceived and reported. Because child self-reports were not collected, the present study cannot distinguish whether these associations primarily reflect children’s actual well-being, parents’ perceptions of their children’s well-being, or a combination of both. Consequently, the findings should be interpreted as relating to parent-perceived child HRQoL rather than objective child HRQoL. Findings from this community-based sample suggest that parent-proxy child HRQoL is associated with parental psychosocial functioning. Parental mental HRQoL emerged as the strongest independent correlate of parent-proxy child HRQoL. However, given the exclusive use of parent-proxy reports and the convenience-based sampling strategy, these findings should be interpreted cautiously, as shared method variance, rater-related effects, and limited generalizability may have contributed to the observed associations. Further multi-informant and longitudinal studies, including child self-reports and more diverse populations, are needed to clarify the relationships between parental psychological functioning and children’s health-related quality of life.

## Figures and Tables

**Table 1:** Demographic characteristics of parents.

Characteristics	*n*	%
Gender		
Male	27	11.2
Female	215	88.8
Parental education		
Secondary	53	21.9
Tertiary	101	41.7
MSc/PhD	88	36.4
Age		
≤40	89	36.8
≥41	153	63.2
Marital status		
With partner/spouse	217	89.7
Without partner/spouse	25	10.3
Children with chronic illness		
Yes	23	9.5
No	219	90.5

**Table 2:** Descriptive statistics for PCS, MCS, PedsQL, TAI and SAI.

Scale	Mean ± SD	Cronbach’s α
SF-12		
PCS	42.85 (±5.59)	0.789
MCS	36.92 (±6.98)	0.781
PedsQL Total	66.40 (±8.05)	0.837
Physical Functioning	66.94 (±10.13)	0.738
Emotional Functioning	65.70 (±10.40)	0.744
Social Functioning	66.67 (±11.48)	0.697
School Functioning	65.99 (±11.80)	0.646
Psychosocial Health	66.12 (±8.71)	0.789
STAI		
TAI	41.80 (±5.40)	0.949
SAI	43.00 (±13.40)	0.914
RCADS		
RCADS Depression	3.59 (±3.18)	0.813
RCADS Anxiety	4.99 (±4.36)	0.823

**Table 3:** Bivariate analyses between demographic characteristics, study scales/subscales, and PedsQL.

Variable	U/H-Test or Spearman’s ρ	PedsQL *p*-Value
Gender ^a^	2311	0.084
Parental education ^b^	1075.3	0.005
Age group ^a^	5794.50	0.048
Marital status ^a^	1592	0.022
Children with chronic illness ^a^	2340	0.575
PCS	0.042	0.677
MCS	0.213	0.031
TAI	−0.204	0.004
SAI	−0.314	<0.001
RCADS Depression	−0.369	<0.001
RCADS Anxiety	−0.322	<0.001

^a^ *p*-value was calculated using the Mann–Whitney U-test. ^b^ *p*-value was calculated using the Kruskal–Wallis H-test. Categorical variables were compared using the Mann–Whitney U or Kruskal–Wallis tests. Continuous variables were examined using Spearman’s rank correlation coefficient (ρ).

**Table 4:** Multivariate regression analysis of predictors of PedsQL.

Variables	B	Standard Error	β	t	*p*	95% CI
Constant	95.922	13.698	—	7.002	<0.001	68.553, 123.291
Parental education	−2.944	1.433	−0.254	−2.055	0.044 *	−5.807, −0.081
Age	0.514	2.099	0.033	0.245	0.807	−3.680, 4.708
Marital status	−5.673	4.644	−0.153	−1.221	0.226	−14.952, 3.606
MCS-12	0.344	0.144	0.315	2.395	0.020 *	0.06, 0.62
TAI	1.503	3.697	0.059	0.406	0.686	−5.884, 8.890
SAI	−0.476	2.034	−0.039	−0.234	0.816	−4.540, 3.588
RCADS depression	−7.303	5.055	−0.261	−1.445	0.153	−17.403, 2.797
RCADS anxiety	−2.695	5.652	−0.089	−0.477	0.635	−13.988, 8.598

* *p* < 0.05.

## Data Availability

The data presented in this study are available on request from the corresponding author due to privacy and ethical restrictions.
